# Risk Factors Associated with Maternal Postpartum Hospital Readmission: A Systematic Review

**DOI:** 10.3390/nursrep16070218

**Published:** 2026-06-26

**Authors:** Haichao Huang, Mingzhu Wu, Huaqiong Zhou, Weixin Jiang, Paul Porter, Kym Jones, Xiang Wang, Phillip Roy Della

**Affiliations:** 1 School of Nursing, Tianjin University of Traditional Chinese Medicine, Tianjin 301617, China; huanghc@tjutcm.edu.cn (H.H.); jiangweixin@stu.tjutcm.edu.cn (W.J.); 2 School of Graduate, Tianjin University of Traditional Chinese Medicine, Tianjin 301617, China; wumingzhu@stu.tjutcm.edu.cn; 3 Curtin School of Nursing, Curtin University, Perth, WA 6102, Australia; h.zhou@curtin.edu.au; 4 Perth Children’s Hospital, Nedlands, WA 6009, Australia; 5 Department of Paediatrics, Joondalup Health Campus, Perth, WA 6027, Australia; paul.porter@curtin.edu.au; 6 Faculty of Health Science, Curtin University, Perth, WA 6102, Australia; 7 Partnerships for Health Innovation (PHI) Research Group, Joondalup Health Campus, Perth, WA 6027, Australia; 8 Department of Gynaecology and Obstetrics, Joondalup Health Campus, Perth, WA 6027, Australia; kym.jones@curtin.edu.au; 9 Tianjin Jinnan District Health Commission, Tianjin 300350, China; wangxiang2225@163.com

**Keywords:** maternal readmission, postpartum period, postpartum readmission, review, risk factors

## Abstract

**Background**: Maternal postpartum hospital readmissions represent profound implications for maternal health outcomes and potential gaps in quality of maternal care. **Objective**: This study aims to synthesise evidence on risk factors for maternal postpartum hospital readmissions within 42 days of discharge following birth hospitalisation. **Methods**: An electronic database search utilised CINAHL, EMBASE (Ovid), and MEDLINE for relevant studies published from 1 January 2010 to 30 June 2024. The studies that investigated the prevalence and risk factors for 42-day postpartum maternal readmission and reported risk estimates, published in English, were included. The risk of bias was assessed using the Newcastle–Ottawa Scale (NOS) for case-control studies and cohort studies. The PRISMA guidelines were followed in reporting this review. The review protocol was registered on PROSPERO (CRD42023442269). **Results**: A total of 7758 articles were retrieved, ultimately including 60 studies. The rate of maternal postpartum readmissions varied from 0.1236‰ to 26%. Significant risk factors were extracted and categorised into five groups: maternal demographic and socio-economic factors; behavioural and lifestyle factors; health institution structural factors; obstetric and delivery characteristics; as well as maternal morbidity The most frequently cited risk factors which contributed to maternal postpartum hospital readmissions were age, race/ethnicity, substance use, caesarean delivery, length of maternal hospital stay, premature birth, and all maternal morbidities, especially mental health disorders, severe maternal morbidity, and hypertensive disorders of pregnancy. **Conclusions**: This systematic review identified complex and diverse risk factors associated with maternal postpartum hospital readmissions within 42 days after discharge following birth hospitalisation. This helps our understanding of the risk factors and the strength of association with maternal postpartum hospital readmissions. Future research should develop a multidimensional risk assessment framework to guide clinical practice in adopting holistic individualised approaches for postpartum risk evaluation, thereby reducing readmission rates and improving maternal health outcomes.

## 1. Introduction

Postpartum hospital readmission (PPHR) is defined as a new mother’s readmission to the hospital within a specific time frame following childbirth [1,2]. Although the timeframe for tracking postpartum readmissions is variable, 42 days after delivery discharge is commonly used, as this period is a critical time for the mother’s physical and psychological recovery, as well as when many postpartum complications are likely to arise.

The incidence of PPHR, accounting for a substantial portion of maternal morbidity and mortality, has received global attention, which represents profound implications for maternal health outcomes and potential gaps in the quality and safety of maternal care [3]. In addition, maternal postpartum readmission within 42 days of initial discharge disrupts the crucial maternal–neonatal bond during the sensitive period [4], exacerbates the financial burden on the healthcare system [5], and causes more significant family function impairment [6].

The World Health Organization (WHO) emphasises the importance of quality postnatal care for the health and well-being of women after childbirth and their newborns. It highlights the global focus on reducing postpartum readmissions as a measure of quality care [7]. Identifying risk factors associated with PPHR can inform the development of evidence-based clinical practice guidelines to improve discharge preparedness for women who need additional care or monitoring, thereby reducing the likelihood of maternal readmission. Current research has identified risk factors for PPHR from multiple perspectives. However, the existing literature lacks a comprehensive systematic overview of the risk factors associated with PPHR. This paper aimed to review the characteristics of the included studies and synthesise the identified risk factors for PPHR within 42 days after delivery.

## 2. Materials and Methods

The review protocol was preregistered with the PROSPERO International Prospective Register of Systematic Reviews (code: CRD42023442269) and conducted following the Preferred Reporting Items for Systematic Reviews and Meta-Analysis (PRISMA) 2020 Statement [8] (checklist in Table S1).

### 2.1. Data Sources and Search Strategy

An electronic database search was conducted using CINAHL, EMBASE (Ovid), and MEDLINE with the key search terms, spanning from 1 January 2010 to 30 June 2024. We further examined the references of the included studies to identify relevant additional literature. A complete search strategy is provided in Table S2.

### 2.2. Eligibility Criteria

We defined the inclusion criteria in our review as follows: (i) primary research studies assessed the prevalence and risk factors of postpartum maternal readmission; (ii) PPHR measurement within 30 days (defined as 28–32 days) or 42 days after delivery; when multiple time were examined, only extracted data for 30-day or 42-day readmissions; (iii) study design stated clearly and reported risk estimates, including *p*-values, odds ratios (ORs)/relative risks (RRs)/hazard ratios (HRs), and 95% confidence interval (CI) or raw data could be calculated in an appropriate format; (iv) published in peer-reviewed journals; and (v) in English language with full text available.

Our exclusion criteria were as follows: (i) outcomes mixed PPHR with complications and/or Emergency Department (ED) visits; (ii) having two or more readmissions within the 30- or 42-day span of the trace, or maternal readmission after stillbirth; (iii) experimental design evaluating the effect of an intervention on readmission; (iv) studies focused on maternal readmission during the COVID-19 pandemic (due to the phased impact on PPHR); (v) conference abstract-only references.

### 2.3. Records Screening and Study Selection

All records identified using our search strategy were exported to EndNote. Duplicate articles were removed. Two reviewers (HH and HZ) independently screened all titles and abstracts for potential inclusion. Then, the full-text manuscripts of all the potentially eligible studies were reviewed against the inclusion criteria. Only a handful of disagreements between the reviewers were resolved by discussion. A hand search of the reference lists of all potential articles was conducted to identify any further relevant articles.

### 2.4. Data Extraction

The data were manually extracted from the eligible studies by the first reviewer (HH) and independently reviewed for accuracy and consistency by the third reviewer (HZ). Discrepancies were resolved through discussion or consultation with the last author. There were three discrepancies: the exact effect estimate, the definition of follow-up period, and whether a specific confounder was reported. All discrepancies were resolved through re-examination of the original articles and discussion with the last author. Finally, a consensus was reached among the three authors. Data were extracted using a data extraction form that included study characteristics, examined variables and identified risk factors. Study characteristics included study setting, population, sample size, timing of data collection, study design, data source, readmission rate and statistical analysis used to identify risk factors. Examined variables or confounding factors and significant risk factors were extracted. No automation tools were used in the data collection process.

### 2.5. Quality Assessment

The included studies were assessed for quality using the Newcastle–Ottawa Scale (NOS) quality assessment scale for case-control and cohort studies (checklist in Table S4). The evaluation focused on three aspects, selection of study groups, comparability, and outcome (case-control studies)/exposure (cohort studies), including eight items, with a maximum score of 9 points. Scores of quality assessment were grouped into three categories: low (0–3), moderate (4–7), and high (≥8). Disagreements were addressed and resolved by all the authors.

### 2.6. Data Preparation and Synthesis

Effect measures were extracted from the original publications, including readmission rate (proportions) and risk factors (*p*-values or ORs, RRs and HRs with 95% confidence intervals). If these were not reported, we attempted to calculate them from the available data. If the necessary data were not available in the publication, we contacted the corresponding authors by email to request it. Meta-analysis was not feasible for this review due to substantial clinical heterogeneity across study populations, risk factors, and follow-up periods examined for readmission risk. Accordingly, all included studies were eligible for a narrative synthesis. Studies were grouped and synthesised by the type of risk factors examined to facilitate a structured comparison.

## 3. Results

**Figure 1:**
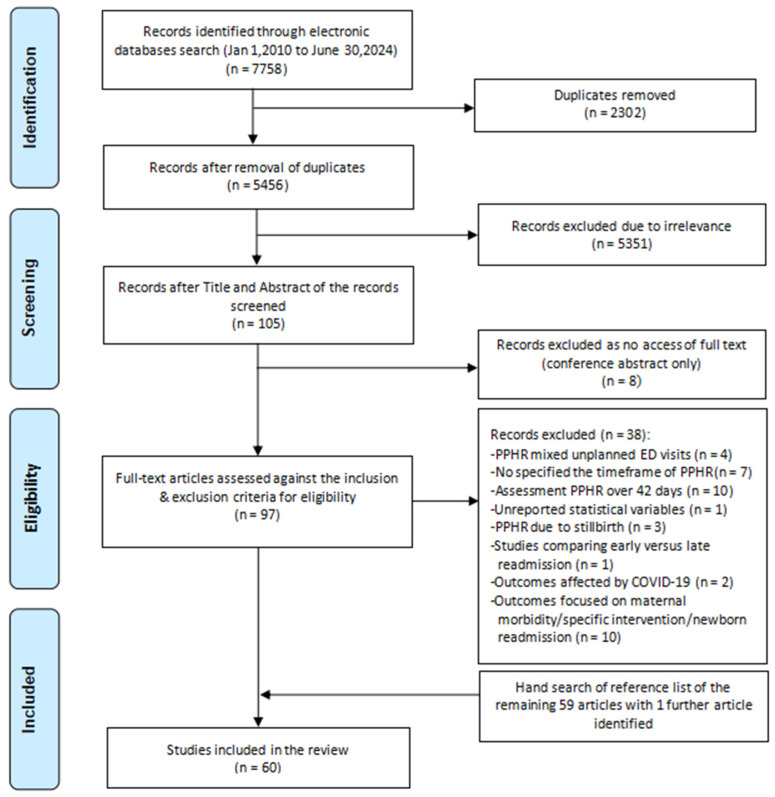
Flow chart for the search and study selection process (PRISMA).

The initial electronic database search from 1 January 2010 to 30 June 2024 generated 7758 records. After removing duplicates and screening titles and abstracts, 97 references were reviewed in full text and assessed for eligibility. One additional article was identified during a hand search of the reference lists [9]. As a result, 60 studies were included in the systematic review. Figure 1 illustrates the search result and selection process. The studies excluded at the full-text screening stage, along with the reasons for exclusion, are presented in Table S3.

### 3.1. Characteristics of the Included Studies

Table 1 summarises the characteristics of the 60 included studies. Nearly 80% of the included studies (n = 46) were conducted in the United States of America (USA), three in the United Kingdom (UK), two each in Israel, and one each in Australia, Belgium, Canada, China, Egypt, Italy, Iran, Korea, and Poland. Forty included studies used a standard 42-day PPHR measurement, including one study [10] that further measured early readmission (7 days) and later readmission (8–42 days), while the remaining 20 included studies measured readmission over periods ranging from 28 to 32 days; these were assigned to the 30-day readmission category.

The majority of studies (n = 55) employed retrospective data collection, while five studies involved a prospective research design. All the included studies were of moderate to high quality (Table 2 and Table 3). Forty-three studies collected data from multiple sites, compared to 17 from single centres. The data retrieval period ranged from 80 days [11] to 22 years [12]. In particular, nine included studies [12,13,14,15,16,17,18,19,20] collected data over 10 years. Sample sizes varied from 82 [21] to 39,212,104 [20] postpartum women. Across the 60 included studies, the reported readmission rate varied from 0.1236‰ [22] to 26% [21]. Eleven of the 60 included studies reported risk-predictive performance models. Six studies employed a multivariable logistic regression model [23,24,25,26,27,28], one study used a generalised mixed linear model [15], two used Cox proportional hazard models [16,29], one study applied gradient boosted trees to generate a model [30], and the remaining one [31] used an internal–external cross-validation (IECV) approach. All eleven identified predictive models in the studies demonstrated moderate to high performance, with C-statistics ranging from 0.73 [27] to 0.90 [26].

### 3.2. Examined Variables and Significant Risk Factors

The examined variables and significant risk factors extracted from the 60 included studies are summarised in Table 4. Each study’s examined variables ranged from one to twenty-nine. Seven studies [12,32,33,34,35,36,37] reported inconclusive outcomes. The statistically significant risk factors were categorised into two PPHR measurements (30-day and 42-day readmissions) and presented under five subheadings: maternal demographic and socio-economic factors, maternal behavioural and lifestyle factors, health institution structural factors, obstetric and delivery characteristics, and maternal morbidity.

#### 3.2.1. Demographic and Socio-Economic Factors

A total of eight maternal sociodemographic variables were associated with PPHR. The variables included age, race/ethnicity, marital status, education level, types of insurance, income level [22,38,39,40,41,42,43,44], place of residence [39,45] and living arrangement [46]. The three most frequently cited risk factors were age, race/ethnicity, and insurance type.

**Table 1:** Characteristics of the 60 included studies.

Reference	Medical Condition	Outcome Measures	Study Design	Data Source	Sample Size	Age	Follow-Up Period	Proportion Readmitted	Data Analysis
Gibson,2024 [47] USA	All births with recorded blood pressure 24 h prior to discharge	42-Day	Retrospective	50 US states, 119 health systems; electronic health records	1,265,766	32	2017–2023	BP (mmHg) <120/80: 1% ≥120/80: 1.3% ≥130/80: 2% ≥140/90: 3.2% ≥150/100: 4.5% ≥160/110: 5%	Univariate regression
Lui, 2024 [39] USA	All cause	30-Day	Retrospective	4 States’ inpatient databases	2,073,489	30.3 ± 6.0 (readmission); 29.5 ± 5.7 (without)	2015–2020	1.7%	Generalised linear mixed models
Mitro, 2024 [48] USA	Hypertensive disorders	42-Day	Retrospective	Kaiser Permanente Northern California (KPNC) electronic health records	57,254	<25: 16.6% 25–29: 26.1% 30–34: 33.2% ≥35: 24.1%	2012–2018	3.0%	Cox proportional hazards regression models
O’Carroll, 2024 [36] UK	Caesarean patients	32-Day	Prospective	Patient-reported outcomes from 107 obstetric units within the NHS in the UK	1000	32–34	Baseline: a 2-week in Oct 2021; Follow-up: Day 1, Days 28–32	Asian: 7% Black: 4% White: 6%	Chi-square test
Turner, 2024 [49] UK	All cause	30-Day	Retrospective	3 maternity services in England; hospital administration systems	64,250	<20 (3.27%) 20–24 (15.35%) 25–29 (28.58%) 30–34 (31.31%) 35–39 (17.15%) 40+ (4.35%)	2015–2020	4.5%	Multilevel logistic regression
Zhu, 2024 [35] USA	HDPs	42-Day	Retrospective	Electronic medical records, a secondary analysis of STAMPP-HTN program	1477	30 (24,35)	2018–2020	14.9%	Chi-square test
Bronner, 2023 [50] USA	HDPs	42-Day	Retrospective Cohort Study	A single academic institution; electronic medical records	545	15–45	2019–2021	8.33% (Extended monitoring group); 4.08% (preintervention group)	Multivariable analysis
Brown, 2023 [10] USA	All patients, focus on mental health conditions	42-Day	Retrospective Cohort Study	Administrative database	12,222,654	No specified	2016–2019	>3 or 2 or 1 mental health conditions: 42 d (3.38%, 2.33%, 2.17%); 1–7 d (1.53%, 1.19%, 1.16%); 8–42 d (1.81, 1.14%, 1.01%)	Multivariable logistic regressions
Gesner, 2023 [27] USA	Preeclampsia	42-Day	Retrospective	A public county hospital; medical records	440	No readmission group30.2 ± 7.00 (No PPHR); 33.6 ± 6.31 (PPHR)	2016–2022	10.0%	Multivariate logistic regression; ROC
Kumar, 2023 [51] USA	All deliveries, focus on HDP	30-Day	Retrospective	Multicentre; medical records	9457	26.9 ± 5.9 (PPHR); 27.0 ± 5.9 (No PPHR)	2010–2013	1.7% (All); 3.4% (HDP)	Multivariable logistic regression
Majmundar, 2023 [20] USA	All cause	42-Day	Retrospective	Nation-wide readmission database	39,212,104	28.6 ± 6.3 (with CVD) 28.3 ± 5.9 (No CVD)	2010–2019	1.484%	Multivariable logistic regression
McKee, 2023 [16] USA	All deliveries, focus on deaf or hard of hearing (DHH)	42-Day	Retrospective	Administrative database (PELL)	1,385,003	DHH: 28.8 ± 6.4; non-DHH: 29.7 ± 5.9	1998–2016	1.66% (DHH); 1.11% (No DHH)	Cox proportional hazard models
Lin, 2024 [28] USA	Preeclampsia with severe features	42-Day	Retrospective case–control study	Clinical research data warehouse linked to institutional electronic medical records	348	Readmitted: 34.2 ± 5.7; Controls: 34.3 ± 5.3	2012–2019	NA	Multivariable logistic regression; ROC
Venkatesh, 2023 [31] USA	All deliveries, focus on HDPs	42-Day	Retrospective	2 tertiary care health systems; electronic health records	28,201	30 ± 5.6	2014–2015; 2017–2019	0.9%	Penalised Logistic Regression; internal–external cross validation; c-statistic
Cozzi, 2023 [52] USA	All deliveries, focus on pre-delivery anaemia	42-Day	Retrospective Cohort Study	A tertiary hospital; medical records	18,357	26.3 ± 5.9 (PPHR); 27.7 ± 6.0 (No PPHR)	2013–2018	4.9% (PPHR, anaemia); 3.4% (PPHR, no-anaemia);	Multivariable logistic regression
Girsen, 2022 [18] USA	All delivery	29-Day	Retrospective	State-wide database	5,248,746	29.9 ± 6.6 (0–6 d PPHR); 28.3 ± 6.2 (7–29 d PPHR)	2007–2018	0.45%	Multivariable logistic regression
Kawakita, 2022 [25] USA	Focused on Emergency Severity Index	42-Day	Retrospective	An urban hospital; electronic medical records	439 of 5860 women had CD presented to ED	30.2 ± 6.2 (Level 1–2) 30.3 ± 6.0 (Level 3) 28.7 ± 5.9 (Level 4–5)	2012–2018	22%	Multivariate logistic regression; AUC
Panzer, 2022 [11] USA	All deliveries, focus on expedited postpartum discharge	42-Day	Retrospective	2 affiliated hospitals; electronic medical records	1358	No specified	2019–2020	3.2% (All, Routine), 4.0% (All, Expedited discharge); 4.3% (HDP, Routine), 20.7% (HDP, Expedited discharge)	Logistic regression stratified analysis
Wang, 2022 [17] Taiwan	Pregnancy with hyperglycaemia	30-Day	Retrospective	Nation-wide electronic database	1,830,511	No specified	2008–2017	Approx. 2.4% to 14.8%	ANOVA
Yoselevsky, 2022 [53] USA	Postpartum eclampsia	42-Day	Retrospective	Nation-wide database	1,590,563 deliveries	30.1 ± 6.3 (PPHR); 28.8 ± 5.8 (No PPHR)	2016	0.1%	*t*-test
Bae, 2021 [14] USA	All deliveries, focus on sickle cell disease	30-Day	Retrospective	3 State databases	6,911,916	28.60 ± 5.9	2007–2014	4.4% (sickle cell disease); 2.6% (sickle cell trait); 1.5% (non-sickle cell disease)	Multivariable logistic regression
Black, 2021 [54] USA	All deliveries	30-Day	Retrospective	Administrative database	165,444	31.98 ± 5.98 (PPHR); 32.45 ± 5.90 (No PPHR)	2016	1.17%	Multivariable logistic regression
Bruce, 2021 [55] USA	HDP	42-Day	Retrospective	Administrative database (KPNC); electronic medical records	7151	34.0 ± 5.2 (PPHR); 31.6 ± 5.6 (No PPHR)	2018	4.43%	Multivariable logistic regression
Christenson, 2023 [21] USA	Preeclampsia	42-Day	Prospective Cohort Study	A tertiary hospital; observation	82 women	≥18 years	2017–2018	26%	*t*-test
Cioci, 2021 [56] USA	All deliveries, focus on VTE	30-Day	Retrospective	Administrative data (NRD)	2,790,579	>14 years	2014	0.053%	Multivariable logistic regression
Decker, 2021 [38] USA	Epilepsy	30-Day	Retrospective	Administrative data (NRD)	38,518 with epilepsy vs. 8,136,335 controls	27.28 (Epilepsy) 28.16 (Control)	2015–2017	2.4%	Multivariable logistic regression
Ghaffari, 2021 [37] Iran	Elective caesarean	42-Day	Prospective	A tertiary general hospital; medical records	294	29.49 ± 4.07 (24 h discharge); 29.46 ± 3.82 (48 h discharge)	2018–2019	2.04%	*t*-test
Hoffman, 2021 [30] USA	All deliveries, focused on HDP	42-Day	Prospective	A large tertiary institution; electronic medical records	25,855	30.69 ± 5.66 (PPHR); 29.45 ± 5.46 (No PPHR)	2015–2019	1.2% (Derivation); 1.4% (Validation)	Gradient Boosted Trees; AUC
Matas, 2021 [57] USA	SMM	42-Day	Retrospective	Administrative data (NRD)	6,193,852	≥15	2016–2017	1.4% (Overall); 4.9% (with SMM)	Multivariate logistic regression
McLaren, 2021 [15] USA	Focused on postpartum preeclampsia	42-Day	Retrospective case-control study	A single, high-volume urban hospital; medical records	807	33 (PPHR); 29 (No PPHR)	2010–2020	No specified	Mixed linear modelling; AUC
Mukhtarova, 2021 [23] USA	All delivery, focused on HDP	42-Day	Retrospective	A tertiary hospital; electronic database	24,917	32 ± 6.1 (PPHR); 30 ± 5.4 (No PPHR)	2009–2015	0.45%	Logistic regression; AUC
Patterson, 2021 [32] Australia	large volume transfusion	42-Day	Retrospective	State-wide database	1011	No specified	2006–2010	9.9% (tertiary); 8.2% (non-tertiary)	Multivariable regression
Sakai-Bizmark, 2021 [46] USA	All deliveries, focus on postpartum living arrangement	42-Day	Retrospective	State-wide databases	1,109,785	27.7 ± 6.83 (homeless); 29.3 ± 6.1 (non-homeless)	2009–2014	1.4% (Homeless); 1.6% (non-homeless)	Multivariable regression
Srebnik, 2021 [12] Israel	Infection	42-Day	Retrospective	A tertiary hospital; electronic database	58,174	27.09 ± 5.38 (PPIM) 28.161 ± 5.46 (No PPIM)	2005–2017	No specified	*t*-test
Stamilio, 2021 [24] USA	All deliveries, focused on HPD	42-Day	Retrospective	A tertiary care medical centre; electronic health records	14,503 cohort (58 PPHR, matched 232 No-PPHR)	30.74 ± 6.26 (PPHR); 29.74 ± 5.95 (No PPHR)	2012–2015	0.4%	Multivariate logistic regression; AUC
Valent, 2021 [19] Italy	All deliveries	42-Day	Retrospective	A tertiary hospital; administrative health database	20,756	Median 31 (PPHR); Median 32 (No PPHR)	2000–2018	0.48%	Multivariate logistic regression
Chornock, 2020 [58] USA	HPD	42-Day	Retrospective	3 regional hospitals; electronic perinatal database	4317	31.3 ± 7.0 (PPHR); 28.0 ± 6.8 (No PPHR)	2009–2016	1.5%	Multivariate logistic regression
Eriksen, 2020 [59] USA	Class III obesity	42-Day	Retrospective	6 hospitals; electronic dataset	20,556 (overall); 904 (Class III obesity)	Not specified	2012–2017	2.2%	Multivariate logistic regression
Foeller, 2020 [9] USA	All deliveries, focus on Sepsis	42-Day	Retrospective	State-wide database, linked hospital data	1,880,758	28.2 ± 6.6 (PPHR); 28.3 ± 6.4 (No PPHR)	2008–2011	0.01%	Multivariable logistic regression
Girsen, 2020 [60] USA	All deliveries, focus on SMM	42-Day	Retrospective	Electronic databases	2,413,943	29.4 ± 6.8 (PPHR with SMM); 27.8 ± 6.7 (PPHR without SMM); 28.4 ± 6.3 (No PPHR)	2008–2012	1.01% (Overall) 1.8% (SMM) 0.83% (No SMM)	Chi-square test; ANOVA
Lui, 2020 [61] USA	All deliveries, focused on opioid use disorder (OUD) by CD	30-Day	Retrospective	3 state-wide electronic databases	2,425,527	29.66 ± 6.08	2007–2014	4.2% (PPHR with); non-OUD 1.9%	Multivariate logistic regression
Kopeć-Godlewska, 2020 [45] Poland	All deliveries, focused on infection	30-Day	Retrospective	29 hospitals; National Health Fund database	68,894	No specified	2013–2014	1.7% (Overall); 0.8% (Infection)	Multivariate logistic regression
Nam, 2020 [29] Korea	Focused on SMM	42-Day	Retrospective	National health database	90,035	≥15	2003–2013	0.95%	Cox proportional hazard model
Patel, 2020 [62] USA	All deliveries	42-Day	Retrospective	A tertiary hospital; electronic medical records	8589	30.1 ± 6.1 (PPHR) 29.3 ± 6.1 (No PPHR)	2015–2019	2.4%	Chi-square; Student’s *t*-test; ANOVA
Plusquin, 2020 [34] Belgium	All deliveries	42-Day	Retrospective	A tertiary hospital; electronic health database	7160	29 ± 5.5 (01/2010–05/2025); 30 ± 7.5 (06/2010–11/2015)	2010–2015	1.17%	Multivariate logistic regression
Salemi, 2020 [40] USA	Focused on drug users	42-Day	Retrospective	Administrative data (NRD)	11 million	18–49	2010–2014	Drug user 5.46%; non-drug 1.4%	Multivariate logistic regression
Fitzpatrick, 2019 [13] UK	CD	42-Day	Retrospective	Administrative database	74,043	No specified	2002–2015	2.92% (elective repeat CD); 2.49% (vaginal birth after previous CD)	Logistic or modified Poisson regression
Lima, 2019 [63] USA	All deliveries, focus on heart disease (HD)	42-Day	Retrospective	Nation-wide database	15,273,247	28.9 ± 0.06 (HD) 28.3 ± 0.03 (No HD)	2010–2014	5.2% (HD); 1.4% (No HD)	Multivariable logistic regression
Miller, 2019 [22] USA	All deliveries, focus on postpartum stroke	30-Day	Retrospective	Administrative database	17,215,614	25.4 (no stroke readmission); 30.0 (stroke readmission)	2010–2014	0.01236‰	Poisson regression
Wagner, 2019 [41] USA	All deliveries, focus on preeclampsia	30-Day	Retrospective	Administrative database	4,999,993	28.74 ± 5.99	2007–2014	1.2%	Multivariate logistic regression
Zmora, 2019 [64] Israel	All deliveries, focus on retained placenta	42-Day	Retrospective	A medical centre; electronic medical records	101,185	29.0 ± 5.7 (Retained placental fragments exposed); 28.3 ± 5.6 (with normal 3rd stage)	2005–2014	0.33%	Multivariate logistic regression
Clapp, 2018 [44] USA	All deliveries	30-Day	Retrospective	Administrative database	3,164,429 (California); 1,890,802 (Florida); 2,257,360 (New York):	27.13–29.60	2004–2011	1.15–1.57%	Chi-square test
Clapp, 2017 [3] USA	All deliveries	42-day	Retrospective	Administrative database	5,949,739	28.6 ± 7.5 (2004–2005); 28.6 ± 7.8 (2006–2007); 28.9 ± 8.2 (2008–2009); 29.4 ± 8.5 (2010–2011)	2004–2011	1.8–2.1%	Logistic regression
Bayoumi, 2016 [33] Egypt	CD	42-Day	Prospective	A maternity hospital; medical records and survey	2998	No specified	2012–2014	3.7% (24 h); 3.4% (72 h)	*t*-test
Clapp, 2016 [43] USA	All deliveries	42-Day	Retrospective	State-wide databases	5,949,739	35.8 (readmitted); 28.2 (no-readmitted)	2004–2011	1.93%	Logistic regression
Hirshberg, 2016 [65] USA	Preeclampsia	28-Day	Retrospective control case study	A single institution; medical records	99	28.0 ± 7.0 (preeclampsia and readmitted); 26.7 ± 6.9 (preeclampsia and no-readmitted)	2005–2009	25.3%	Multivariable logistic regression
Aseltine, 2015 [42] USA	All deliveries	30-Day	Retrospective	Administrative database	243,453	28.7 ± 6.1 (VD); 30.5 ± 6.0( CD)	2005–2012	0.88% (VD); 1.57% (CD)	Logistic regression
Stamilio, 2014 [66] USA	Obesity	28-Day	Retrospective cohort study	Secondary analysis based on the data from a randomised controlled trial	585	27.7 ± 6.2 (obesity); 27.9 ± 5.1 (extreme obesity)	2008–2010	5.0%	Logistic regression
Macdonald, 2015 [67] Canada	All deliveries, focus on HIV	30-Day	Retrospective control case study	State-wide linked database	1,133,505	18–49	2002–2011	2.8% (HIV); 1.1% (No-HIV)	Generalised estimating equations
Larsen, 2012 [26] USA	Focus on preeclampsia	28-Day	Retrospective control case study	A single institution; medical records	153	disease group: 28.5; control group: 26.3	1998–2003	No specified	Logistic regression; AUC

Notes: CD: caesarean delivery; CVD: cardiovascular disease; HD: heart disease; OUD: opioid use disorder; VD: vaginal delivery.

**Table 2:** Overview of bias assessment results using the Newcastle–Ottawa scale for included studies: cohort studies (55).

Reference	Selection				Comparability	Outcome			Score
	Representativeness of the Exposed Cohort	Selection of the Non-Exposed Cohort	Ascertainment of Exposure	Demonstration that Outcome of Interest was not Present at Start of Study	Comparability of Cohorts on the Basis of the Design or Analysis	Assessment of Outcome	Follow-Up Was long Enough for Outcomes to Occur	Adequacy of Follow-Up of Cohorts	
Gibson, 2024 [47]	√	√	√	√	√√	√	√	√	9
Lui, 2024 [39]	√	√	√	√	√	√	√		7
Mitro, 2024 [48]	√	√	√	√	√	√	√	√	8
O’Carroll, 2024 [36]	√	√	√	√	√√	√	√		8
Turner, 2024 [49]	√	√	√	√	√	√	√	√	8
Zhu, 2024 [35]		√	√	√	√	√	√		6
Bronner, 2023 [50]		√	√	√	√√	√	√		7
Brown, 2023 [10]	√	√	√	√	√√	√	√		8
Christenson, 2023 [21]		√	√	√	√	√	√	√	7
Gesner, 2023 [27]		√	√		√√	√	√		6
Kumar, 2023 [51]	√	√	√	√	√√	√	√		8
Majmundar, 2023 [20]	√	√	√	√	√√	√	√		8
McKee, 2023 [16]	√	√	√	√	√√	√	√		8
Venkatesh, 2023 [31]		√	√	√	√√	√	√		7
Cozzi, 2023 [52]		√	√	√	√√	√	√		7
Girsen, 2022 [18]	√	√	√	√	√√	√	√		8
Kawakita, 2022 [25]		√	√	√	√√	√	√		7
Panzer, 2022 [11]	√	√	√	√	√√	√	√		8
Wang, 2022 [17]		√	√	√	√√	√	√		7
Yoselevsky, 2022 [53]	√	√	√	√	√	√	√		7
Bae, 2021 [14]	√	√	√	√	√√	√	√		8
Black, 2021 [54]	√	√	√	√	√	√	√		8
Bruce, 2021 [55]	√	√	√	√	√	√	√		7
Cioci, 2021 [56]	√	√	√	√	√√	√	√		8
Decker, 2021 [38]	√	√	√	√	√√	√	√		8
Ghaffari, 2021 [37]		√	√	√	√	√	√	√	7
Hoffman, 2021 [30]		√	√	√	√	√	√	√	7
Matas, 2021 [57]	√	√	√	√	√√	√	√		8
Mukhtarova, 2021 [23]		√	√	√	√√	√	√		7
Patterson, 2021 [32]	√	√	√	√	√√	√	√		8
Sakai-Bizmark, 2022 [46]	√	√	√	√	√√	√	√		8
Srebnik, 2021 [12]		√	√	√	√	√	√		6
Stamilio, 2021 [24]		√	√	√	√√	√	√		7
Valent, 2021 [19]		√	√	√	√√	√	√		7
Chornock, 2021 [58]		√	√	√	√√	√	√		7
Eriksen, 2020 [59]	√	√	√	√	√√	√	√		8
Foeller, 2020 [9]	√	√	√	√	√√	√	√		8
Girsen, 2020 [60]	√	√	√	√	√	√	√		7
Lui, 2020 [61]	√	√	√	√	√√	√	√		8
Kopeć-Godlewska, 2020 [45]	√	√	√	√	√√	√	√		8
Nam, 2020 [29]	√	√	√	√	√√	√	√		8
Patel, 2020 [62]		√	√	√	√	√	√		6
Plusquin, 2020 [34]		√	√	√	√√	√	√		7
Salemi, 2020 [40]	√	√	√	√	√√	√	√		8
Fitzpatrick, 2019 [13]	√	√	√	√	√√	√	√		8
Lima, 2019 [63]	√	√	√	√	√√	√	√		8
Miller, 2019 [22]	√	√	√	√	√√	√	√		8
Wagner, 2019 [41]	√	√	√	√	√√	√	√		8
Zmora, 2019 [64]		√	√	√	√√	√	√		7
Clapp, 2018 [44]	√	√	√	√	√	√	√		7
Clapp, 2017 [3]	√	√	√	√	√√	√	√		8
Bayoumi, 2016 [33]		√	√	√	√√	√	√	√	8
Clapp, 2016 [43]	√	√	√	√	√√	√	√		8
Aseltine, 2015 [42]	√	√	√	√	√	√	√		7
Stamilio, 2014 [66]		√	√	√	√	√	√		6

Notes: The checkmark (√) denotes compliance with each item of the Newcastle–Ottawa Scale for quality evaluation of cohort studies. The total NOS score ranges from 0 to 9, with higher scores indicating lower risk of bias.

**Table 3:** Overview of bias assessment results using the Newcastle–Ottawa scale for included studies: case-control studies (5).

Reference Number	Selection				Comparability	Exposure			Score
	Adequate case Definition	Cases were Representative	Selection of Controls	Definition of Controls	Comparability of Cases and Controls Based on Design or Analysis	Ascertainment of Exposure	Same Method of Ascertainment for Cases and Controls	Non-Response rate	
Lin, 2024 [28]	√	√	√	√	√√	√	√		8
McLaren, 2021 [15]		√	√	√	√	√	√	√	7
Hirshberg, 2016 [65]	√		√	√	√√	√	√		7
Macdonald, 2015 [67]		√	√	√	√√	√	√		7
Larsen, 2012 [26]	√		√	√	√√	√	√		7

Notes: The checkmark (√) denotes compliance with each item of the Newcastle–Ottawa Scale for quality evaluation of cohort studies. The total NOS score ranges from 0 to 9, with higher scores indicating lower risk of bias.

Maternal age was cited as a significant factor associated with 30-day readmission by five studies [22,38,49,54,56], and 42-day readmission by thirteen studies [10,15,23,27,29,30,40,43,53,55,58,60,64]. The overwhelming majority of included studies identified advanced maternal age as a risk factor of readmission, among which four, further stratified by age, indicated that women in the group aged 40 years and above had a higher rate of PPHR compared to those under 40. One study [58] even indicated that the risk of 42-day readmission due to hypertension increased by 1.44 times for every 5-year age increment. However, a study involving participants with severe maternal morbidity found that women aged 20–30 years had a higher risk of 42-day readmission in comparison to those over 30 years old [29].

Race/ethnicity was identified as a risk factor for PPHR, irrespective of whether a 30-day [26,39,41,42,44,54,66] or 42-day [15,24,28,30,43,55,58,60] readmission measurements were used. Eight studies reported women of Black race were more likely to be readmitted (OR = 1.22–2.09) compared with others. Furthermore, two studies cited that the non-Hispanic Black race increased 42-day readmission [58,60], while another study [24] reported the Hispanic black race was 2.14 times more likely to be readmitted within 42 days. Three studies (one used 30-day readmissions and two within 42-day readmission) cited that African Americans were more likely to experience PPRH for hypertensive disorders of pregnancy (HDP) or postpartum preeclampsia [26] (Larsen, 2012), examining a 30-day window and in two studies using a 42-day window [28,30]. While three studies found that Hispanic ethnicity was associated with an increased risk of 30-day readmission [39,41,42], one study reported a lower likelihood of 30-day readmission (OR = 0.80) [54].

Type of health insurance was also reported as a factor associated with PPHR: private insurance has been consistently cited as having the lowest readmission rate compared to Medicare, Medicaid, and other types of health insurance in both 30-day [38,39,41,42,44,56] and 42-day [23,40,43,60] readmissions.

#### 3.2.2. Behavioural and Lifestyle Factors

Behavioural and lifestyle factors were reported to be associated with PPHR. Four studies cited that women with substance use were linked to an increased incidence of PPHR within 30 days [41,61] or 42 days [40,43] compared to nonusers (OR = 1.27–2.86). Specifically, one study highlighted that multiple drug users were associated with a higher risk of 42-day readmission than single-category users [40]. Additionally, tobacco use was identified as another behavioural and lifestyle risk factor for readmission in studies using either a 30-day [44] or a 42-day time window [43,53].

#### 3.2.3. Health Institution Structural Factors

Hospital structural characteristics, including hospital type, teaching status, hospital delivery volume, and midwifery staffing levels, were identified to be associated with increased odds of PPHR.

Hospital type as a predictive factor of PPHR was reported. Studies using a 30-day (*p* = 0.009) [44] and a 42-day (OR = 0.898) [43] readmission time window, respectively, reported community hospital designation as a protective factor for readmission. One study found women who delivered in tertiary hospitals were at a high risk of 42-day readmission (HR = 1.55) [29]. One study found that index admission at a for-profit hospital was associated with nearly a twofold increase in the odds of venous thromboembolism readmission within 30 days compared with non-profit hospitals [56].

**Table 4:** Examined variables of the 60 included studies and the significant risk factors associated with PPHR.

**Reference Number**		**[47]**	**[39]**	**[48]**	**[36]**	**[49]**	**[35]**	**[50]**	**[10]**	**[27]**	**[51]**	**[20]**	**[16]**	**[28]**	**[31]**	**[52]**	**[18]**	**[11]**	**[17]**	**[53]**	**[14]**	**[54]**	**[55]**	**[21]**	**[56]**	**[38]**	**[37]**	**[30]**	**[57]**	**[15]**	**[23]**
**Examined Variables (n=)**		1	12	15	1	7	1	1	1	13	21	1	1	7	9	21	22	16	2	12	1	29	14	13	11	6	1	13	1	16	12
**Significant Risk Factors (n=)**		1	4	1	0	3	0	1	1	2	5	1	1	3	6	1	11	1	1	8	1	29	7	1	5	6	0	5	1	3	10
**Demographic and** **socio-economic factors**	age					✓				✓					✓					✓		✓	✓		✓	✓		✓		✓	✓
	race/ethnicity		✓											✓								✓	✓					✓		✓	
	types of insurance		✓																						✓	✓					✓
	income level		✓																							✓					
	place of residence		✓																												
	living arrangement																														
	marital status																														✓
	education level																														
**Behavioural and lifestyle factors**	substance use																														
	tobacco use																			✓											
**Health institution structural factors**	hospital type																								✓						
	teaching status																									✓					
	delivery volume																														
	midwifery staffing					✓																									
**Obstetric and delivery characteristics**	mode of delivery					✓					✓				✓		✓			✓		✓	✓		✓			✓			✓
	LOS																✓	✓				✓			✓	✓					✓
	gestational age																✓			✓			✓								✓
	parity														✓																
	multiple gestation																			✓		✓									
	adverse history																														
	conception method																														
	length of labour																														
**Maternal morbidities**	mental health								✓								✓					✓									
	SMM																✓					✓							✓		
	comorbidities *										1	1				1	2		1	2	1	11				1		1			2
	complications **										2						3					9									1
	HDP ***	1		1				1		1				2	2		1			1		2	3	1				1		1	1
	others												✓		✓																
**Reference Number**		**[32]**	**[46]**	**[12]**	**[24]**	**[19]**	**[58]**	**[59]**	**[9]**	**[60]**	**[61]**	**[25]**	**[45]**	**[29]**	**[62]**	**[34]**	**[40]**	**[13]**	**[63]**	**[22]**	**[41]**	**[64]**	**[44]**	**[3]**	**[33]**	**[43]**	**[65]**	**[42]**	**[66]**	**[67]**	**[26]**
**Examined variables (n=)**		1	11	2	7	14	11	10	7	12	11	8	6	12	12	19	14	4	1	11	18	14	26	16	1	23	21	11	5	10	8
**Significant risk factors (n=)**		0	1	0	4	1	2	1	6	10	1	1	2	4	4	0	14	1	1	9	15	11	23	1	0	18	2	11	5	2	4
**Demographic and socio-economic factors**	age						✓			✓				✓			✓			✓		✓				✓					
	race/ethnicity				✓		✓			✓											✓		✓			✓		✓	✓		✓
	types of insurance									✓							✓				✓		✓			✓		✓			
	income level																✓			✓	✓		✓			✓		✓			
	place of residence												✓																		
	living arrangement		✓																												
	marital status																														
	education level									✓																					
**Behavioural and lifestyle factors**	substance use										✓						✓				✓					✓					
	tobacco use																						✓			✓					
**Health institution structural factors**	hospital type													✓									✓			✓					
	teaching status												✓							✓											
	delivery volume							✓													✓		✓								
	midwifery staffing																														
**Obstetric and delivery characteristics**	mode of delivery								✓	✓					✓			✓		✓	✓		✓			✓				✓	
	LOS					✓														✓	✓							✓			
	gestational age								✓													✓	✓			✓					
	parity									✓				✓	✓							✓									
	multiple gestation									✓													✓			✓					
	adverse history																					✓	✓			✓					
	conception method																					✓									
	length of labour																										✓				
**Maternal morbidities**	mental health																✓						✓			✓					
	SMM									✓				✓																	
	comorbidities *									✓					✓		✓		✓	✓	✓	✓	✓	✓		✓		✓	✓		✓
	complications **								✓						✓					✓	✓	✓	✓			✓		✓		✓	
	HDP ***				✓												✓			✓	✓		✓			✓	✓	✓	✓		✓
	others											✓									✓							✓			

* Comorbidities include asthma, anaemia, sickle cell disease, diabetes mellitus, thyroid disorders, breast disorders, neurologic disorders, seizure, cardiovascular diseases, gall bladder diseases, liver diseases, renal diseases, immune system disorders, HIV, and comorbidity index. ** Complications include vomiting, complications of anaesthesia, antepartum/postpartum haemorrhage, injury/trauma, retained placenta, thrombotic events, and infection. *** HDP includes preeclampsia, mild preeclampsia, severe preeclampsia/eclampsia, pregnancy-related hypertension, chronic hypertension, pulmonary hypertension, unspecified hypertension, maximum SBP, maximum DBP, MAP (Mean Arterial Pressure), extended blood pressure monitoring, BP 24 h prior to discharge, received or usage of antihypertensive medications.

Hospital teaching status was identified to be associated with 30-day readmission in three studies. One study reported that the distribution of hospital teaching status differed significantly between women with and without 30-day stroke readmission (*p* = 0.007), with a higher proportion of 30-day stroke readmissions occurring in metropolitan teaching hospitals. Consistently, another study reported that among women with epilepsy, those delivered at non-metropolitan hospitals had lower odds of 30-day readmission compared to those at metropolitan teaching hospitals (OR = 0.60) [38]. However, one study found that the risk of readmission due to infection was higher among women who gave birth at lower-referral-level hospitals than at teaching hospitals (OR = 2.81) [45].

Hospital delivery volume was identified as a factor of postpartum readmission in three studies. One study reported that hospitals with higher delivery volume (Quartiles 1–2) had higher odds for 30-day readmission rates than lower-volume hospitals (notably Quartile 4) (*p* < 0.001) [44]. Whereas another study found that women who delivered in hospitals with the lowest quartile of delivery volume were more likely to be readmitted up to 30 days postpartum (*p* < 0.0001) [41]. In addition, one study reported women with pre-pregnancy class III obesity delivering in low-prevalence institutions had a significantly increased odds of 42-day readmission than in high-prevalence hospitals (OR = 15.20) [59].

One study [49] reported that below-average midwife staffing was associated with more PPHR.

#### 3.2.4. Obstetric and Delivery Characteristics

In the studies included, there were eight risk factors related to obstetric and delivery characteristics that contributed to PPHR, including mode of delivery, length of initial hospital stays for delivery (LOS), gestational age, parity, multiple gestation, history of previous pregnancy and delivery, conception methods (natural conception or assisted reproductive technology), and length of labour.

The mode of delivery was identified as the most frequently reported risk factor for PPHR. Women who had a caesarean delivery were consistently identified as having a higher risk of being readmitted within either 30 days or 42 days (OR = 1.28–3.13), while only one study found no association between caesarean and hypertension readmission within 30 days [65].

The association between LOS and PPHR was reported. Nine studies identified that prolonged LOS, especially greater than 3 or 4 days, had higher odds of PPHR. Four studies examined the association between early discharge and postpartum readmission, with three reporting no significant impact on 30-day or 42-day readmission [34,35,39]. Another study reported that the risk for PPHR did not differ significantly between expedited (<2 d after vaginal birth and <3 d after caesarean birth) and routine discharges (≥2d after vaginal birth and ≥3 d after caesarean birth) but found that expedited discharge was associated with significantly high risk for 42-day readmission among women with preeclampsia, gestational hypertension, and chronic hypertension (OR = 5.78) [11].

Gestational age was reported as a significant predictor of PPHR. Despite the heterogeneity in readmission definitions (ranging from all-cause readmission to condition-specific readmission, such as for HDP or sepsis, most studies consistently reported the association between premature birth and readmission (*p* < 0.01/0.001, OR = 1.45–7.30), demonstrating an inverse relationship between gestational age and the likelihood of PPHR. One study specifically reported that women who delivered before 28 weeks had more than a 7-fold increased risk for readmission with sepsis within 42 days of delivery [9]. Only one study cited that gestational age at delivery was not significantly associated with 42-day readmission for HDP [55].

Parity as a risk factor for PPHR was reported. Compared with multiparous women, primiparous women were associated with an increased risk of 42-day readmissions (*p* < 0.01–0.05; OR = 1.31–1.59) [29,60,62,64]. However, one study reported that women who had previously given birth were at a higher risk of 42-day readmissions due to HDP (*p* < 0.001) [30].

Five studies consistently reported multiple gestation as associated with an increased risk of PPHR compared with singleton gestation (*p* < 0.001–0.01; OR = 1.20–1.55). One study cited that women who had undergone assisted reproductive technology were more likely to be admitted within 42 days after delivery (OR = 1.73) [64].

Three studies referred to an adverse obstetric history (including previous pregnancy losses and prior caesarean delivery) as a risk factor for PPHR in a subsequent pregnancy, of which one used 30-day readmissions [44] Clapp, 2018 and two used 42-day readmissions [40,64]. One study mentioned conception achieved through assisted reproductive technology was associated with an increased risk of 42-day readmission compared to spontaneous conception (OR = 1.73) [64]. One study reported that prolonged labour duration was a risk factor for 30-day readmission in women with postpartum hypertension (OR = 1.06) [65].

#### 3.2.5. Maternal Morbidities

(1) Mental Health Disorders

Mental health disorders were examined across six different studies, with three studies examining 30-day readmissions [18,44,54] and the others measuring 42-day readmissions [10,40,43]. Consistent findings from the six studies showed that mental health conditions increased the risk of postpartum readmission (OR = 1.09–2.54). Furthermore, one study found that greater numbers of mental health conditions than a single type of mental disorder were associated with increased readmission rates [10].

(2) Severe Maternal Morbidity (SMM)

Severe maternal morbidity (SMM) was consistently associated with a higher readmission rate across studies that used either a 30-day [18,54] or a 42-day [29,57,60] readmission measurement. Moreover, a study [18] (Girsen, 2022) reported that the risk estimates for SMM-related readmission were broadly similar between early (≤6 days) (OR = 3.46) and late (7–29 days) (OR = 3.27) readmission.

(3) Pre-existing Comorbidities

Ten studies reported that pregnant women with pre-existing diabetes mellitus (DM) or gestational diabetes mellitus (GDM) had an increased risk of PPHR (OR = 1.18–1.85), of which six used 30-day readmissions [17,18,41,42,44,54], and four used a 42-day measurement [23,43,53,64]. One study further indicated that women with GDM had a modest decrease in the 30-day readmission rate compared to those with known DM and newly diagnosed DM (*p* = 0.046).

Five studies referenced the increased risk of PPHR associated with neurologic system diseases. Two studies reported that women with neurologic disorders had higher odds of 30-day readmission (OR = 1.55–2.57) [41,42]. One study cited that epilepsy was associated with a 1.9-fold increase in the odds of postpartum readmission within 30 days [38]. Two studies reported that seizure disorder also increased the risk of readmission, of which one used 30-day readmissions (*p* < 0.001) [44] and the other used 42-day readmissions (OR = 1.989) [43].

Four studies consistently reported that the coexistence of thyroid diseases during pregnancy increased the risk of 30-day or 42-day readmission (OR = 1.06–1.40) [18,43,44,54]. Three studies demonstrated that cardiovascular diseases (*p* < 0.0001 or OR = 2.36–3.84) [20,40,63], asthma (OR = 1.28–1.39) [43,44,54], and anaemia before delivery (OR = 1.18–1.35) [41,52,54] were associated with higher odds of 30- or 42-day readmission. Two studies each reported an increased risk of PPHR associated with renal disease (OR = 1.55–2.83) [40,54], sickle cell disease (OR = 1.27–2.32) [14,40], and immune system disorders (OR = 1.87–2.23) [40,54].

One study each mentioned that individuals with HIV (OR = 2.53) [67], breast disorder (OR = 2.26) [54], gall bladder disease (OR = 2.18) [54], liver disease (OR = 1.51) [54], and bleeding disorders (OR = 1.47) [18] before birth hospitalisation were associated with an increased risk of 30-day readmission, respectively.

In addition, three studies identified the Comorbidity Index as a risk factor correlated with heightened rates of 30-day readmission [22,41,42], and one study reported a similar association with the Emergency Severity Index within 42-day readmission [25]. One study indicated that women with deaf or hard-of-hearing (DHH) were more likely to be readmitted within 42 days after delivery compared to those without DHH (OR = 1.84) [16].

(4) Obstetrics Complications

Various types of infectious complications were reported as risk factors for PPHR. Two studies, one using a 30-day readmission window and the other a 42-day window, both reported chorioamnionitis (OR = 1.10–2.30) [9,51] as a risk factor for PPHR. Similarly, urinary tract infections were identified as a risk factor in two studies (one within 30 days and the other within 42 days) (OR = 1.19–2.50) [9,54]. One study reported that pneumonia increased the risk of 42-day PPHR (OR = 5.7) [9]. Several other studies reported unspecified infections as a risk factor for 30-day readmissions [22,41,42,44,54]. One study [18] cited that sepsis during the index admission was associated with PPHR within 30 days (OR = 4.92). One study reported no significant association between leukocyte count at admission for labour or pre/postpartum difference and maternal readmission [12].

Eight studies referred to peripartum haemorrhagic complications, which were associated with an increased risk of postpartum readmission. Two studies reported that women with antepartum haemorrhage due to placenta previa faced a higher likelihood of being readmitted (OR = 1.14–1.40) [43,54]. Seven studies identified that women who experienced intrapartum and/or postpartum haemorrhage were more likely to be readmitted [9,18,23,44,54,62,64]. One study specifically indicated that primiparous women with blood loss ≥1000 mL during delivery were more susceptible to being readmitted within 42 days after delivery (*p* < 0.05) [62]. One study further reported that intrapartum and/or postpartum haemorrhage associated with a decrease in haemoglobin ≥3 g% or blood product transfusion was associated with 2.73- and 1.54-fold increases in 42-day readmission, respectively [64].

Three studies explored the correlation between injury/trauma and PPHR, yielding inconsistent results. Two studies reported that specific injury or trauma, including pelvic and peroneal trauma, uterine rupture, higher-order laceration and operative injury, contributed to an increased risk for 30-day readmission [44,54]. In contrast, one study reported no significant association between perineal trauma and 30-day readmission [18].

Additionally, one study each reported excess vomiting of pregnancy (OR = 1.28–1.38) [54], thrombotic event (*p* < 0.001) [44] and retained placenta with 3rd stage of labour removal of placental fragments by manual uterine revision (OR = 1.44) [64] were associated with PPHR.

(5) Hypertensive Disorders of Pregnancy

Prenatal diagnosis of HDP as a primary driver of PPHR was identified. Studies using either 30-day readmissions [22,41,42,44,51] or those using a 42-day readmission [43,53] reported hypertensive disorders (not specifying the subtype) as a risk factor for readmission (OR = 1.37–2.29 or *p* < 0.05). Similarly, preeclampsia in pregnancy [18,24,26,30,40,41] (OR = 1.26–10.91, or *p* < 0.001), gestational hypertension [24,26,40,43,44] (OR = 1.65–3.44, or *p* < 0.001) and pre-existing hypertension [22,30,40,54] (OR = 1.59–3.67, or *p* < 0.05) were consistently reported as risk factors for PPHR, irrespective of whether a 30-day or 42-day readmission was used. Two studies reported that women who developed postpartum hypertension or hypertension diagnoses at discharge were at a higher risk of 42-day readmission (*p* < 0.01) [21,23]. Particularly, one study highlighted that pulmonary hypertension raised the risk of 42-day readmission by 7.26 times [40]. One study reported that a blood pressure reading exceeding 130/80 mmHg within the 24 h before discharge was associated with an increased risk of 42-day readmission [47]. A retrospective cohort study found that extending monitoring to 36 h before discharge with a strict BP target (<150/100 mmHg) did not reduce 42-day readmission for severe preeclampsia in women with a history of HDP [50]. In addition, two studies (one using 30-day readmissions [65] and the other 42-day readmissions [55]) revealed that antihypertensive medications were a protective factor against PPHR in women with hypertension (OR = 0.23–0.80). One study reported that the use of two or more antihypertensive agents was a protective factor against 42-day readmission (OR = 0.53) [48].

## 4. Discussion

This systematic review synthesised risk factors associated with maternal readmission within 42 days after delivery. Sixty studies were reviewed, and all identified risk factors were grouped into five categories: demographic and socio-economic factors; behavioural and lifestyle factors; health institution structural factors; obstetric and delivery characteristics; and maternal morbidity. There is a significant disparity in the prevalence of PPHR. All the included studies were of moderate to high quality, with no low-quality studies identified, indicating that the overall quality of evidence is acceptable. The risk factors associated with PPHR are complex and heterogeneous, reflecting the varied reasons for maternal readmission and the research focus.

Our study showed through included studies that advanced age, Black or Hispanic race, public insurance and poor income level were associated with PPHR in terms of maternal demographic and socioeconomic factors, and substance use was the main risk factor in terms of behavioural and lifestyle factors. Increasing maternal age, particularly over 40 years, was directly correlated with SMM [68], making them more likely to have caesarean sections due to medical indications such as placenta previa and foetal distress, which would experience a slower recovery due to decreased physical resilience and pre-existing health conditions, leading to a higher likelihood of readmission if complications arise during recovery. Racial and ethnic disparities in outcomes of PPHR were significant, particularly for Black and Hispanic women, who were more likely to live in socioeconomically disadvantaged areas [69] and had less access to private insurance, further limiting their ability to receive adequate care [70]. Additionally, Black and Hispanic women were more likely to undergo caesarean deliveries [71], which was associated with increased risk odds of poor outcomes in a more extended recovery period and more postpartum complications. Special attention to individuals with advanced age, as well as Black and Hispanic women, expanding the coverage of public insurance services for women with low socioeconomic status would reduce the risk odds associated with PPHR. Potential detrimental effects for the mother and her baby can be minimised or averted through the validity of universal screening for substance use during pregnancy and further treatment as well as comprehensive care throughout pregnancy for those who test positive in the screening [72]. Referring perinatal patients to a substance use provider in a timely manner is also critical for improving maternal health outcomes and reducing postpartum readmission [73].

Postpartum readmission rates are associated with four main health institution structural factors, including tertiary hospitals, teaching hospitals, delivery hospital volume, and average midwife staffing. However, a previous study reported that less than 1% of the variation in readmission rates was attributable to hospital factors, suggesting that hospital factors did not have a profound impact on PPHR. There should be an increasing focus on the quality of care at hospitals in the days before and after childbirth, rather than just on the type of hospital [44].

Our review highlights the significance of obstetric and delivery characteristics. Three notable risk factors are caesarean delivery, longer LOS, and preterm. Nineteen studies included found that caesarean delivery increased the rate of PPHR. Previous research evidence indicated that caesarean damaged the endometrial and muscle layer of the uterus, and subsequent pregnancies after caesarean were prone to complications such as placenta previa and placental adhesion, which affected the process of postpartum recovery and led to increased readmission. Surgical site infection is also a major contributor to readmissions after caesarean [74,75]. A longer LOS implies that there may be complex health issues for the parturient, which could potentially prolong the postpartum recovery and increase the rate of PPHR. However, a shorter LOS (early discharge) does not increase the risk of PPHR in the included studies, except for women with medical conditions [11]. The majority of included studies demonstrated the correlation between premature birth and PPHR. Women who experience preterm birth are at a higher risk of developing postpartum infection [76], often involving caesarean, and experiencing higher levels of anxiety and depression [77], which can lead to additional medical complications and increase the odds of PPHR. Therefore, promoting vaginal delivery over caesarean and providing comprehensive postpartum care for women with caesarean deliveries or premature birth can help reduce the risk of complications leading to readmission. Moreover, discharge decisions should be based on the mother’s actual health condition, rather than on a routine schedule, to reduce PPHR rates.

Our study results also show that both pre-existing comorbidities and complications during pregnancy and the postpartum period can increase the rate of PPHR. In particular, mental health disorders, SMM and HDP merit particular attention as risk factors reported as associated with readmission.

The studies included consistently reported strong associations between mental health conditions during the perinatal period and risk of PPHR, which is similar to previous research findings among individuals with mental health disorders [78,79]. Individuals with mental health conditions face an increased risk of adverse perinatal outcomes during the postpartum period, such as inability to perform self-care, self-harm or suicide, and a greater propensity for substance abuse [80,81]. In addition, women with perinatal comorbidities or complications are more likely to suffer from perinatal mental disorders, which further exacerbate adverse effects [82]. Given the severe adverse outcomes of perinatal mental health, it is reasonable to suggest the need for multifaceted efforts. During the prenatal and intrapartum period, health staff may reasonably consider providing targeted education, psychological support and skill guidance, especially for high-risk pregnant women, helping expectant mothers reduce stress and trauma brought on by pregnancy and delivery. A more important practical implication is to provide ongoing support during the postpartum period, including comprehensive discharge instructions, timely screenings for postpartum depression, providing counselling to alleviate anxiety, encouraging family support and social interaction, as well as continuity of care through conventional follow-ups and methods supported by information technology.

Severe maternal morbidity (SMM) is defined by the Centres for Disease Control and Prevention (CDC) as an index of 18 life-threatening conditions associated with both short- and long-term maternal morbidity [83]. The leading indicators of SMM at readmission are blood transfusions in response to postpartum haemorrhage, hysterectomy, multiple chronic comorbidities, preeclampsia, temporary ventilatory, pulmonary oedema, acute heart failure and sepsis [83,84,85]. Based on the available evidence, health staff may consider conducting a thorough assessment of SMM that extends beyond the prenatal period into the broader postpartum period to identify emerging conditions and take effective measures to reduce its impact. Furthermore, integrated multidisciplinary care for high-risk individuals, improved patients’ recognition of early warning signs through standardised education programs, effective discharge readiness, and ongoing postpartum care as needed may help to improve maternal safety and reduce PPHR.

Our study’s findings underscore the heightened risk of PPHR among women with a prenatal diagnosis of HDP, particularly those identified with preeclampsia. The results were consistent with the existing literature, which reports that HDP, especially preeclampsia, was a significant predictor of maternal mortality as well as postpartum readmission [86]. The increased vulnerability of these women to postpartum readmission may be attributed to the type and severity of HDP, as well as the effectiveness of blood pressure management. In particular, elevated blood pressure within 24 h of discharge was identified as a significant predictor of PPHR, emphasising the importance of close blood pressure monitoring in the postpartum period and the potential benefits of initiating antihypertensive treatment at lower thresholds to reduce the risk of readmission [65]. Therefore, a reasonable clinical implication is that postpartum care can be tailored to the specific risks associated with HDP, with a focus on early identification, aggressive medication management, and continuity of care through remote monitoring and a telehealth approach [87] to prevent HDP-related readmissions.

Most identified risk factors were similar for both the 30-day and 42-day readmissions. The major strengths of our study were the rigorous methods used to synthesise and interpret a wide range of evidence on maternal postpartum readmissions. We searched three major medical databases, including CINAHL, EMBAS, and MEDLINE, and predefined strict study selections, ensuring that comprehensive and relevant studies were considered. In addition, the large number and substantial heterogeneity of the original studies make synthesising evidence on risk factors for maternal readmission quite complex. To our knowledge, this is the first systematic review of risk factors for maternal postpartum readmission within 42 days of discharge following birth hospitalisation.

We acknowledge that this study’s limitations include the biases implicit in estimating the effect size. The mixing of high- and moderate-quality studies across risk factors prevented quality-stratified comparisons, thereby limiting our ability to directly assess the impact of study quality on effect sizes. The focus of the included studies markedly differed, from all-cause readmissions to those for specific reasons or populations. This diversity in readmission criteria led to substantial variation in the reported rates and risk factors, making a meta-analysis or further subgroup analyses unfeasible. Furthermore, given that all primary studies are observational, the reported factors should be viewed as predictive indicators rather than causal agents. Consequently, although some factors (such as private insurance, mental health conditions, severe maternal morbidity) showed consistent evidence supporting them as readmission risk factors, they should be interpreted with caution in clinical practice.

## 5. Conclusions

Our findings highlight the multifaceted and complex nature of the risk factors associated with maternal postpartum readmission within 42 days of delivery. Consistently cited predictors include Black race, low-income level, non-private insurance, substance use disorders, multiple gestation, previous adverse obstetrics history, mental health disorders, severe maternal morbidities, HDP, as well as all comorbidities and complications related to pregnancy and childbirth. The results highlight the significant contribution of racial and social determinants of health, as well as any maternal morbidity, to postpartum readmissions. The solutions to reduce PPHR, firstly, should consider focusing on specific high-risk populations such as women with Black race, mental health issues, SMM or HDP, which may be more effective than efforts targeting the general population. For high-risk populations, in addition to enhancing management and monitoring during birth hospitalisation, providing extended postpartum care and lengthening the duration of postpartum visits should also be considered. There is an imperative need to develop and standardise a comprehensive discharge plan to improve readiness for discharge, including a transition checklist and structured communication tools.

Future research is warranted to develop risk stratification tools or risk prediction models to accurately identify high-risk postpartum populations for targeted interventions. Educational programs, including training simulations, should be developed to improve healthcare providers’ ability to recognise women at higher risk of readmission following delivery. As comorbidities contribute significantly to PPHR, early intervention that improves women’s health should be implemented during the period of pre-pregnancy and interpregnancy.

## Data Availability

All data analysed in this review are included within the manuscript, Supplementary Materials and referenced articles.

## Supplementary Materials

The following supporting information can be downloaded at: https://www.mdpi.com/article/10.3390/nursrep16070218/s1, Table S1: PRISMA Checklist 2020; Table S2: Full search strategy; Table S3: Full texts excluded with reasons; Table S4: NEWCASTLE–OTTAWA quality assessment scale.

## Public Involvement Statement

No public involvement in any aspect of this research.

## Guidelines and Standards Statement

This systematic review was conducted and reported in accordance with the Preferred Reporting Items for Systematic Reviews and Meta-Analyses (PRISMA 2020) guidelines [8].

## Abbreviations

The following abbreviations are used in this manuscript:

DHH	Deaf or hard-of-hearing
DM	Diabetes mellitus
GDM	Gestational diabetes mellitus
HDP	Hypertensive disorders of pregnancy
PPHR	Postpartum hospital readmission
SMM	Severe maternal morbidity
