# Supplementary material for: Risk Factors Associated with Maternal Postpartum Hospital Readmission: A Systematic Review
Source: Nurs Rep. 2026 Jun 26;16(7):218. doi: 10.3390/nursrep16070218 (PMC13414673; doi:10.3390/nursrep16070218)
Supplement: Supplementary file 1 [file nursrep-16-00218-s001.zip › Table S2_Full Search Strategy.pdf]

Supporting Information File S2: Full Search Strategy

Ovid MEDLINE(R) and Epub Ahead of Print, In-Process, In-Data-Review & Other Non-Indexed Citations, Daily and Versions(R) <1946 to June 30, 2024>

#	Searches
**1**	exp Patient Readmission/
**2**	rehospitali*.tw.
**3**	(readmission* or re-admission* or readmit*).tw.
**4**	(hospital adj5 readmission*).tw.
**5**	(unplanned adj5 readmission*).tw.
**6**	(patient adj5 readmi*).tw.
**7**	(repeat* adj5 hospital*).tw.
**8**	1 or 2 or 3 or 4 or 5 or 6 or 7
**9**	exp Postpartum Period/
**10**	(postpartum or post partum or puerper*).tw.
**11**	exp Parturition/
**12**	(birth* or childbirth* or parturi*).tw.
**13**	exp Lactation/
**14**	exp Breast Feeding/
**15**	(lactat* or breastfeeding or breast feeding).tw.
**16**	(postnatal adj5 period).tw.
**17**	exp Postnatal Care/
**18**	((post or after) adj2 delivery).tw.
**19**	((post or after) adj2 (birth* or childbirth*)).tw.
**20**	9 or 10 or 11 or 12 or 13 or 14 or 15 or 16 or 17 or 18 or 19
**21**	8 and 2**0**
**22**	limit 21 to yr="2000 - 2024"

Embase <1980 to 2024 June 30>

#	Searches
1	exp puerperium/
2	(postpartum or "post partum" or puerper*).tw.
3	exp birth/
4	exp childbirth/
5	(birth* or childbirth* or parturi*).tw.
6	exp lactation/
7	exp breast feeding/
8	(lactat* or breastfeeding or "breast feeding").tw.
9	(postnatal adj5 period).tw
10	exp postnatal care/
11	((post or after) adj2 delivery).tw.
12	((post or after) adj2 (birth* or childbirth*)).tw.
13	1 or 2 or 3 or 4 or 5 or 6 or 7 or 8 or 9 or 10 or 11 or 12
14	exp hospital readmission/
15	rehospitali*.tw.
16	(readmission* or re-admission* or readmit*).tw.
17	(hospital adj5 readmission*).tw.
18	(unplanned adj5 readmission*).tw.
19	(patient adj5 readmi*).tw.
20	(repeat* adj5 hospital*).tw.
21	14 or 15 or 16 or 17 or 18 or 19 or 20
22	13 and 21
23	limit 22 to yr="2000 - 2024"

CINAHL

#	Query
**S22**	S8 AND S21
**S21**	S9 OR S10 OR S11 OR S12 OR S13 OR S14 OR S15 OR S16 OR S17 OR S18 OR S19 OR S20
**S20**	MH "Postnatal Period+"
**S19**	MH "Puerperium"
**S18**	postpartum OR post partum OR puerper*
**S17**	MH "Childbirth+"
**S16**	birth* OR childbirth* OR parturi*
**S15**	MH "Lactation"
**S14**	MH "Breast Feeding+"
**S13**	lactat* OR breastfeeding OR breast feeding
**S12**	postnatal N5 period
**S11**	MH "Postnatal Care+"
**S10**	(post OR after) N2 delivery
**S9**	(post OR after) N2 (birth* OR childbirth*)
**S8**	S1 OR S2 OR S3 OR S4 OR S5 OR S6 OR S7
**S7**	MH "Readmission"
**S6**	rehospitali*
**S5**	re-admission* OR readmission* OR readmit*
**S4**	hospital N5 readmission*
**S3**	unplanned N5 readmission*
**S2**	patient N5 readmit*
**S1**	repeat* N5 hospital*
