# Supplementary material for: Risk Factors Associated with Maternal Postpartum Hospital Readmission: A Systematic Review
Source: Nurs Rep. 2026 Jun 26;16(7):218. doi: 10.3390/nursrep16070218 (PMC13414673; doi:10.3390/nursrep16070218)
Supplement: Supplementary file 1 [file nursrep-16-00218-s001.zip › Table S3_Exclude Through Full text Reading.pdf]

Supporting Information File S3：Full texts excluded with reasons

Excluded article (N=38)	Reason for exclusion
[1] La Rosa M, Jauk V, Saade GR, et al. (2019) Incidence and Risk Factors for Hospital Readmission or Unexpected Visits in Women Undergoing Unscheduled Cesarean Delivery. Am J Perinatol 36: 1115-1119.<br>[2] Leonard SA, Girsen AI, Trepman P, et al. (2024) Early Postpartum Hospital Encounters among Patients with Genitourinary and Wound Infections during Hospitalization for Birth. Am J Perinatol 41: e2017-e2025.<br>[3] Lovgren T, Connealy B, Yao R, et al. (2023) Postpartum medical management of hypertension and risk of readmission for hypertensive complications. J Hypertens 41: 351-355.<br>[4] Janevic, T., Tomalin, L. E., Glazer, K. B., Boychuk, N., Kern-Goldberger, A., Burdick, M., Howell, F., Suarez-Farinas, M., Egorova, N., Zeitlin, J., Hebert, P., & Howell, E. A. (2024). Development of a prediction model of postpartum hospital use using an equity-focused approach. American journal of obstetrics and	Outcome measure of PPHR mixed unplanned Emergency Department visit<br>（n=4）

gynecology, 230(6), 671.e1-671.e10.	
[1] Aziz A, Gyamfi-Bannerman C, Siddiq Z, et al. (2019) Maternal outcomes by race during postpartum readmissions. Am J Obstet Gynecol 220: 484.e481-484.e410.<br>[2] Balhotra K, Roach C, Al-Kouatly HB, et al. (2023) Association of antihypertensive medication at discharge with readmission for postpartum preeclampsia. Am J Obstet Gynecol 228: 747-748.e741.<br>[3] Johnson PD, Duzyj CM, Howell EA, et al. (2019) Patient and hospital characteristics associated with severe maternal morbidity among postpartum readmissions. J Perinatol 39: 1204-1212.<br>[4] Kumar NR, Grobman WA, Barry O, et al. (2021) Evaluating the maternal and perinatal sequelae of severe gestational hypertension. Am J Obstet Gynecol MFM 3: 100280.<br>[5] Panda S, Begley C, Daly D (2016) Readmission following caesarean section: Outcomes for women in an Irish maternity hospital. British Journal of Midwifery 24: 322-328.	Not specified the timeframe for PPHR (n=7)

[6] Parambi A, Davies-Tuck M, Palmer KR (2019) Comparison of maternal and perinatal outcomes in women with super obesity based on planned mode of delivery. Aust N Z J Obstet Gynaecol 59: 387-393.<br>[7] Qureshi. D, al-Jalil. DA, Nadham. R, et al. (2020) Effect of booking status on mode of delivery and postnatal maternal outcome. Bahrain Medical Bulletin.	
[1] Crane DA, Doody DR, Schiff MA, et al. (2019) Pregnancy Outcomes in Women with Spinal Cord Injuries: A Population-Based Study. Pm r 11: 795-806.<br>[2] Ehrenthal DB, Gelinas K, Paul DA, et al. (2017) Postpartum Emergency Department Visits and Inpatient Readmissions in a Medicaid Population of Mothers. J Womens Health (Larchmt) 26: 984-991.<br>[3] Fein A, Wen T, Wright JD, et al. (2021) Postpartum hemorrhage and risk for postpartum readmission. J Matern Fetal Neonatal Med 34: 187-194.<br>[4] Mueller BA, Crane D, Doody DR, et al. (2019) Pregnancy course, infant outcomes, rehospitalization, and mortality among women with intellectual disability. Disabil Health J 12: 452-459.	Outcome of PPHR were measured more than 42days (from 60 days to 2 years) (n=10)

[5] Ouyang L, Cox S, Xu L, et al. (2023) Mental health and substance use disorders at delivery hospitalization and readmissions after delivery discharge. Drug Alcohol Depend 247: 109864.

[6] Pipes GM, Logue TC, Wen T, et al. (2023) Postpartum stroke trends, risk factors, and associated adverse outcomes. Am J Obstet Gynecol MFM 5: 100864.

[7] Stas A, Breugelmans M, Geerinck L, et al. (2023) Implications of a Reduced Length of Postpartum Hospital Stay on Maternal and Neonatal Readmissions, an Observational Study. Matern Child Health J 27: 1949-1960.

[8] Fields JC, Graham HL, Brandt JS, et al. (2023) Risk of postpartum readmission for depression in relation to ischaemic placental disease: a population-based study. EClinicalMedicine 60: 102011.

[9] Wen T, Batista N, Wright JD, et al. (2019) Postpartum readmissions among women with opioid use disorder. Am J Obstet Gynecol MFM 1: 89-98.

[10] Ben Hayoun, D. H., Sultan, P., Rozeznic, J., Guo, N., Carvalho, B., Orbach-Zinger, S., & Weiniger, C. F. (2023).

Association of inpatient postpartum quality of recovery with postpartum depression: A prospective observational study. Journal of clinical anesthesia, 91, 111263. https://doi.org/10.1016/j.jclinane.2023.111263	
[1] Ford JB, Algert CS, Morris JM, et al. (2012) Decreasing length of maternal hospital stay is not associated with increased readmission rates. Aust N Z J Public Health 36: 430-434.	No statistical variables were reported 30 (n=1)
[1] DiTosto JD, Liu C, Wall-Wieler E, et al. (2021) Risk factors for postpartum readmission among women after having a stillbirth. Am J Obstet Gynecol MFM 3: 100345.<br>[2] Wall-Wieler E, Butwick AJ, Gibbs RS, et al. (2021) Maternal Health after Stillbirth: Postpartum Hospital Readmission in California. Am J Perinatol 38: e137-e145.<br>[3] Sweeney, L. C., Reddy, U. M., Campbell, K., & Xu, X. (2024). Postpartum readmission risk: a comparison between stillbirths and live births. American journal of obstetrics and gynecology, 231(4), 463.e1–463.e14.	Maternal postpartum readmission after having a stillbirth (n=3)
[1] Pressman, K., Wellcome, J., Pooran, C., Crousillat, D., Cain, M. A., & Louis, J. M. (2024). Factors associated with early readmission for postpartum hypertension.	Studies comparing early versus late readmission (n=1)

AJOG global reports, 4(2), 100323.	
[1] 23. Kugelman N, Toledano-Hacohen M, Karmakar D, et al. (2021) Consequences of the COVID-19 pandemic on the postpartum course: Lessons learnt from a large-scale comparative study in a teaching hospital. Int J Gynaecol Obstet 153: 315-321.<br>[2] Gulersen M, Husk G, Lenchner E, et al. (2022) The Risk of Readmission after Early Postpartum Discharge during the COVID-19 Pandemic. Am J Perinatol 39: 354-360.	Outcomes of PPHR were affected by COVID-19 epidemic (n=2)
[1] Addae-Konadu, K. L., Wein, L. E., Federspiel, J. J., Hughes, B. L., & Dotters-Katz, S. (2024). Postpartum Pyelonephritis and Risk of Severe Maternal Morbidity. American journal of perinatology, 41(3), 337–342.<br>[2] Alhousseini, A., Farr, C., Ogunyemi, D., Wharton, K., Fawaz, A., Bazzi, N., Andrews-Johnson, T., & Bahado-Singh, R. (2023). Delivery of a Fetus with a Non-Reassuring Status Is Associated with Significant Maternal Morbidity. Gynecologic and obstetric investigation, 88(6), 359–365.<br>[3] Vilda, D., Sutton, E. F., Kothamasu, V. S. S., Clisham, P.	Outcomes focused on maternal morbidity/specific intervention/newborn readmission rather than risk factors of maternal postpartum readmission (n=10)

R., Gambala, C. T., & Harville, E. W. (2024). The risk of perinatal and cardiometabolic complications in pregnancies conceived by medically assisted reproduction. Journal of assisted reproduction and genetics, 41(3), 613–621.

[4] Sharma, C., Sharma, S., & Soni, A. (2024). Subcuticular skin closure at cesarean delivery with poliglecaprone-25 vs polyglactin-910: a randomized controlled trial. American journal of obstetrics & gynecology MFM, 6(2), 101256.

[5] Brown Z, Messaoudi C, Silvia E, et al. (2023) Postpartum navigation decreases severe maternal morbidity most among Black women. Am J Obstet Gynecol 229: 160.e161-160.e168.

[6] Nimal M, Ravel C, Nauleau S, et al. (2023) Relationship between large and small for gestational age and hospital readmission after postpartum discharge: a population-based, data-linkage study. Eur J Pediatr

[7] Meng Z, Zou K, Ding N, et al. (2019) Cesarean delivery rates, costs and readmission of childbirth in the new cooperative medical scheme after implementation of an

episode-based bundled payment (EBP) policy. BMC Public Health 19: 557. 182: 2245-2252.<br>[8] Debost-Legrand A, Rivière O, Dossou M, et al. (2015) Risk Factors for Severe Secondary Postpartum Hemorrhages: A Historical Cohort Study. Birth 42: 235-241.<br>[9] Mardy AH, Siddiq Z, Ananth CV, et al. (2017) Venous Thromboembolism Prophylaxis During Antepartum Admissions and Postpartum Readmissions. Obstet Gynecol 130: 270-278.<br>[10] Robison E, Heyborne K, Allshouse AA, et al. (2017) Implementation of a Risk-Based Heparin Protocol for Postpartum Venous Thromboembolism Prevention. Obstet Gynecol 130: 262-269.	
